# COVID-19 outcomes in HIV patients: A review

**DOI:** 10.1016/j.amsu.2022.103768

**Published:** 2022-05-16

**Authors:** Abdullahi Tunde Aborode, Titilayo Mabel Olotu, O.B. Oyetunde, Abayomi Oyeyemi Ajagbe, Mariam Ayoola Mustapha, Ayah Karra-Aly, Christian Inya Oko

**Affiliations:** a Healthy African Platform, Research and Development, Ibadan, Nigeria; b Student Against COVID-19, Research and Education, USA; c Department of Microbiology, Laboratory of Molecular Biology, Bioinformatics and Immunology, Adeleke University, P. M. B 250, Ede, Osun State, Nigeria; d Healthy Africans Platform, Research and Development, Ibadan, Nigeria; e Department of Anatomy, Faculty of Basic Medical Sciences, College of Health Sciences, Nile University of Nigeria, P.M.B. 900001, Abuja, Nigeria; f National Primary Healthcare Development Agency, Abuja, Nigeria; g Schulich School of Medicine and Dentistry, University of Western Ontario, London, Ontario, Canada; h Institute of Applied Health Research, University of Birmingham, Birmingham, United Kingdom

**Keywords:** COVID-19, HIV, AIDS, Depression, Outcome

## Abstract

The effect of COVID-19 is enormous, and high-risk COVID-19 case arises when underlying infections like diabetes, chronic obstructive pulmonary disease, heart failure, coronary artery disease, or cardiomyopathy are present, and an immunocompromised state such as Human Immunodeficiency Virus (HIV). People living with HIV(PLHIV) may be exposed to severe COVID-19, mostly in areas with poor access to proper care and complex intervention for HIV infection. During the lockdown, those with medical appointments will not access health facilities, which may be detrimental to people living with HIV. Emerging evidence suggests COVID-19 pandemic fear may lead to adverse mental health outcomes and affect preventive behavior. In addition to the stigma and discrimination associated with HIV, COVID-19 is also causing concerns. People with HIV tend to have mental health issues such as depression, anxiety, and post-traumatic stress (PTSD), which can be both a cause and a harmful impact of HIV. Discussed in this research is the effect of the COVID-19 pandemic on HIV patients, their similarities, differences, and urgent attention from healthcare centers to take charge and respond to patients with HIV and other immunosuppressed conditions during the COVID-19 pandemic.

## 1 Background

An outbreak of a deadly coronavirus called COVID-19 has recently hit the globe, causing a pandemic worldwide. Nevertheless, COVID-19 has a wide-ranging impact across people, communities, and societies. COVID-19 is more likely to affect immunocompromised individuals than other populations [1]. On the other hand, HIV (human immunodeficiency virus) has a significant impact worldwide. HIV research and treatment will continue to be impacted by COVID-19, even after COVID-19 becomes milder because COVID-19 affects people living with HIV uniquely [1,2]. A high-risk COVID-19 case arises when underlying infections like diabetes, chronic obstructive pulmonary disease, heart failure, coronary artery disease, or cardiomyopathy are present and an immunocompromised state (weak immune system) [3].

## 2 COVID 19 pandemic IN HIV patients

The World Health Organization (WHO) reports that in the world's poorest countries, there is a disproportionately high rate of HIV infections among existing marginalized populations, such as racial and ethnic minorities and sexual minorities [4].

HIV, on the other hand, acts primarily by destroying immune cells, such as macrophages and CD4^+^ cells, also called helper cells, leaving a person vulnerable to opportunistic infections [1]. Meanwhile, HIV infection leads to Acquired immunodeficiency syndrome (AIDS) when CD4 T cells are reduced below 200 cells per liter of blood. AIDS is defined as having a CD4 T cell count below 200 cells per liter of blood or presenting with an AIDS-defining illness [5].

Putting this aside, people with HIV (PWH) are particularly vulnerable to COVID-19. However, recent research has found that people with HIV may not contract COVID-19 at rapid rates if they receive antiretroviral treatment (ART) [6].

If a PWH is not placed on ART or whose condition is not managed, they are more likely to contract COVID-19 because their immune systems have been compromised, increasing their risk of severe health problems [1]. According to reports, people living with HIV(PLHIV) are not immune-compromised if they adhere to ART [3,7].

Conversely, people living with HIV(PLHIV) may be exposed to severe COVID-19, mostly in areas with poor access to proper care and complex intervention for HIV infection [7]. During the lockdown, those with medical appointments will not access health facilities, which may be detrimental to people living with HIV. COVID-19 is a deadly disease, and those who are HIV-positive should be ensured that the virus is not infecting them [8]. As of April 29, 2021, the World Health Organization (WHO) Global COVID-19 Clinical Data Platform observed over 268 4,12 patients with suspected or confirmed COVID-19 reported by 37 countries [9]. In the WHO African Region, 24 countries provided clinical data on PLHIV. Ninety-six percent (14,914/15,522) of the cases included in the analysis were from South Africa, and ninety-six percent (14,682/15,522) were from Namibia [9]. The majority of PLHIV (5737/15,442) were males, with a mean age of 45.5, 91.8% (8842/9631) taking ART, and 36.2% (5613/15,522) having the severe or critical disease when they were admitted to the hospital. There were 5039 severe cases (5039/5611) and 2187 (2187/5596) of 65 or older [10].

## 3 Similarities and differences between HIV and COVID 19

### 3.1 The transmission mode

HIV belongs to the group of retroviruses known as lentiviruses and the family Retroviridae Lentiviruses [11]. The incubation periods are long, and the illness is long-lasting, making them slow-moving [1]. A significant function of HIV is to deplete macrophages and CD4^+^ cells, which leaves one susceptible to infections caused by opportunistic pathogens [1]. Across the globe, most HIV infections are caused by the HIV-1 subtype. West Africa, however, is the most commonly affected region by the HIV-2 subtype [11].

HIV can be transmitted from person to person via exposure to infected bodily fluids (e.g., blood, semen, vaginal fluids, breastmilk). Transmission occurs most frequently during sexual encounters without condoms, intravenous drug use, occupational exposures, and between mother and infant during pregnancy, delivery, or breastfeeding [1].

Approximately 19 million people have been infected with COVID-19. The virus responsible for this outbreak is the SARS Coronavirus 2 (SARS-CoV-2) [[12], [13], [14], [15]]. Acute respiratory infections, such as SARS-CoV-2, have a short incubation period compared to HIV. A positive-sense single-stranded RNA virus similar to the 2003 global SARS-CoV pandemic, SARS-CoV-2 is a positive-sense single-stranded RNA virus. The SARS-CoV-2 virus belongs to Beta-coronaviruses and the subgroup of arboviruses [12].

In light of recent research, droplets of discharge from the respiratory tract and direct contact with the body are the main transmission routes [16]. According to a study, surfaces can serve as viable reservoirs for virus recurrence [16]. SARS-CoV-2 remains unclear regarding the duration of viral shedding or infection, but the incubation period of 2–14 days is similar to that of other coronaviruses [13]. Since a viral load has not yet been detected for HIV, it cannot be transmitted [17]. In contrast, RNA examination of SARS-CoV-2 does not indicate that the patient is currently infected [18].

### 3.2 Identifying the risks

Based on risk factors, similarities, and differences among COVID-19, it was revealed that failure to follow handwashing and physical distancing recommendations increases the risk of contracting COVID-19 [17,18]. Pre-existing conditions such as hypertension, heart disease, cancer, and diabetes also create a higher risk, as are the elderly and people with pre-existing conditions [19].

In the same way, dangerous behaviors such as sexual contact have been most commonly described as risk factors for HIV. There are also other ways that HIV can be transmitted, such as through blood transfusions or mother-to-child transmission [20].

Specifically, the WHO has established that HIV does affect groups who are marginalized proportionately, and this includes both the racial and ethnic minorities as well as the poor facing social and economic challenges, namely poverty, substance abuse, homelessness, unequal access to health care, and unequal treatment once in the health care system [21].

COVID-19 also doesn't affect minority groups as proportionately as the majority, which is not surprising as there are already existing health disparities [22].

### 3.3 Effects on the human mental health

COVID-19 and HIV share a fear of transmission, despite differences in how the viruses are transmitted [1]. Due to the lack of information regarding the prevention and treatment of COVID-19 at the start of the epidemic, there was a great deal of fear about contracting the infection in the absence of a vaccine or scientifically proven treatments to combat the symptoms.

As a result, fear and anxiety negatively affect mental health and promote disease-related stigma. HIV-related stigma is well recognized, and because of its impact on HIV testing and treatment, numerous research projects have investigated effective interventions to reduce HIV stigma [22].

Emerging evidence suggests COVID-19 pandemic fear may lead to adverse mental health outcomes and affect preventive behavior [22,23]. In addition to the stigma and discrimination associated with HIV, COVID-19 is also causing concerns [24].

People with HIV tend to have mental health issues such as depression, anxiety, and post-traumatic stress (PTSD), which can be both a cause and a harmful impact of HIV.

It does not matter how pandemic fear manifests; it can generally worsen existing mental health disorders and lead to new ones [25]. The elderly, in particular, are likely to experience mental health issues due to social isolation due to COVID-19 [26].

### 3.4 Gap and inequalities in healthcare accessibility

The COVID-19 pandemic highlights the system's inadequacies as it produces health disparities [26]. Those who experience poverty and other systemic stressors are disproportionately burdened with these illnesses. Low-income countries are being adversely affected by the rise in food prices and market stockpiling [27].

Transgender women, commercial sex workers, young women, drug users, and youths often suffer the most significant burden because of their stigmatized or marginalized identities [27]. The number of new HIV infections was accounted for by a third [28].

In addition, immigrants, along with other displaced persons, are at higher risk for contracting infectious diseases such as HIV and COVID-19 [26]. The disease risk and case fatality rates demonstrate this. African American/Black males, who account for 30% of Chicago's population, accounted for approximately 50% of COVID-19 cases and 70% of deaths [29].

## 4 Accessing the effects of COVID-19 on patients with HIV/AIDS

The symptoms that affect HIV patients with COVID-19 are similar to those that affect general populations (cough, fever, malaise, and dyspnea) [7].

A patient with controlled HIV who developed encephalopathy and seizure activity on day 8 of experiencing symptoms with COVID-19 differs from the symptoms of COVID-19 as defined by the WHO [7]. Compared to other studies, this study was not considered an in-depth analysis; however, the patient fully recovered.

Four patients with AIDS followed 32 patients with SARs-CoV-2 and HIV; 90% of the patients recovered fully at the end of their research, 76% died or were hospitalized, and 14% succumbed to acute infection, where the prevalence of SARS-CoV-2 in four patients with AIDS (low CD4 T-cell counts) was higher than that in the overall population, one patient died, three recovered [30].

This implies that people with AIDS may face greater exposure and risk to COVID-19 and pose a severe public health problem [31]. People throughout the world have been affected by the presence of COVID-19. The virus has provoked social distancing business and school closures, affecting daily life significantly. Recognizing the social nature of humans, individuals are finding ways to cope.

Additionally, it is essential to consider how COVID-19 impacts the provision of health care for HIV/AIDS patients (including doctors, social workers, case managers, psychologists, etc.) over time. COVID-19 may have profound effects across all dimensions of PLWH's lives, including biological, psychological, and social aspects.

## 5 HIV/AIDS patients' psychological reaction to COVID-19

A pandemic can worsen mental health problems for people living with HIV due to disruption in continuous care, social isolation, and psychological stress that comes with living through such an event [32]. The stigmatized people with HIV have a greater likelihood of experiencing social isolation. However, COVID-19 prevention measures may exacerbate this experience, resulting in mental health problems [33].

Aside from food insecurity and increased social stigma, COVID-19 is also likely to increase other psychological stressors [33]. Further, psychological stress may reduce medication adherence among persons living with HIV, resulting in inadequate HIV management [29].

### 5.1 Complications of HIV COVID-19 in European countries

In recent years, the SARS-CoV-2 pandemic has disproportionately affected the European region. Many HIV healthcare workers have now turned their attention to the COVID-19 virus, which has a detrimental effect on HIV care and the continuity of antiretroviral therapy. It does not appear that HIV positives or HIV negatives experience different disease courses or rates of COVID-19 infection [34].

About 50% of people living with HIV in European countries are older than 50 and have chronic medical conditions such as chronic lung disease and cardiovascular disease similar to COVID-19 [35]. As a consequence, all patients should be encouraged to stop smoking as well as to get vaccinations against influenza and pneumococcal infections, which have been helpful in preventative manners until now [34].

There was a prevalence of 3.8% for PLWH who contracted COVID-19 [32]^.^ Among 5700 patients with COVID-19 admitted to 12 hospitals in the New York area, 0.8% were infected with HIV [36]. Veterans Aging Cohort Study confirmed 30,891 pregnant women with HIV and 76,745 people with HIV-negative status. There were no differences between the COVID-19 positivity rates (9.7% vs. 10.1%) between the two groups [36].

According to European national HIV/AIDS organizations, there is no evidence that COVID-19 prevalence differs between PLWHIV and the general population. There is no indication that COVID-19 is a problem among PLWHIV [34,36]. Also, PLWH appears to be biologically older by many years, raising the possibility of different thresholds of high risk between PLWH and the general population, which has been a concern in public health [32,36].

## 6 Conclusion

In this research, we describe the impact of the COVID-19 pandemic on HIV patients. We also describe similarities, differences, and risk factors that pose more significant challenges to achieving universal access to healthcare during the pandemic.

The findings suggest that different healthcare centers must take charge and respond to patients with HIV and other immunosuppressed conditions during the COVID-19 pandemic. Infections with HIV or those at risk of contracting it must continue to receive HIV services.

Examples are contraceptives, opiates substitution therapy, sterile needles and syringes, and HIV testing. For vulnerable persons to benefit from COVID-19 services, a targeted approach must be taken to ensure that those left behind are reached, as well as fees must be removed.

## Ethical approval

Not applicable.

## Sources of funding

No funding

## Author contribution

ATA, OTM, OOB study concept or design, data collection, data analysis or interpretation, AOA, MAM and AK writing the paper.

All authors approve the submission

## Trail registry number

1. Name of the registry: Not applicable
2. Unique Identifying number or registration ID: Not applicable
3. Hyperlink to your specific registration (must be publicly accessible and will be checked): Not applicable

## Guarantor

Abayomi Oyeyemi Ajagbe, Department of Anatomy, Faculty of Basic Medical Sciences, College of Health Sciences, Nile University of Nigeria, P.M.B. 900001, Abuja, Nigeria. ORCID: 0000-0002-2110-2626.

## Availability of data and materials

Not applicable.

## Declaration of competing interest

No conflicts of interest.

## Abbreviations

COVID-19: Coronavirus Disease 2019
SARS-CoV2: Severe Acute Respiratory Syndrome – Coronavirus 2
HIV: Human Immunodeficiency Virus
HIV-1: Human Immunodeficiency Virus subtype 1
HIV-2: Human Immunodeficiency Virus subtype 2
PLHIV/PLWH/PWH: People living with HIV
AIDS: Acquired Immune Deficiency Syndrome
ART: Antiretroviral therapy
WHO: World Health Organization?
CD4: Complementary Determining Region 4
HIV/AIDS: Human Immunodeficiency Virus/Acquired Immune Deficiency Syndrome
